# Functional investigation of the *BRCA1 *Val1714Gly and Asp1733Gly variants by computational tools and yeast transcription activation assay

**DOI:** 10.22099/mbrc.2019.33971.1414

**Published:** 2019-09

**Authors:** Fatemeh Yadegari, Leila Farahmand, Rezvan Esmaeili, Tannaz Samadi, Keivan Majidzadeh

**Affiliations:** 1 Genetics Department, Breast Cancer Research Center, Motamed Cancer Institute, ACECR, Tehran, Iran; 2 Recombinant Proteins Department, Breast Cancer Research Center, Motamed Cancer Institute, ACECR, Tehran, Iran

**Keywords:** BRCA1, in silico tools, Functional assay, Yeast

## Abstract

Mutations in the *BRCA1* gene are known to be a major cause of hereditary breast cancer. However, characterizing the point *mutations* associated with cancer in *BRCA1* is challenging because the functional impact of most of them is still unknown. Nowadays, a variety of methods are employed to identify cancer-associated mutations in *BRCA1*. This study is aimed to assess the functional effects of two mutations, Asp1733Gly and Val1714Gly, using a combination of *in* *silico* tools and yeast functional transcription activator assay. Our computational analysis showed that theVal1714Gly mutation was deleterious, while the other one, Asp1733Gly, predicted as neutral. *Also using* yeast functional transcription activator assay, *we found that the Asp1733Gly mutation *displayed similar ability with positive controls. In contrast, the Val1714Gly mutation completely abrogated transcriptional activity in the yeast. These results *suggested *that Val1714Gly and Asp1733Gly can be classified as pathogenic and benign mutations for the BRCA1, respectively.

## INTRODUCTION

*BRCA1* mutations are in close association with hereditary breast and ovarian cancers [-]. The International Agency for Research *on* Cancer (*IARC*) database uses *an integration of personal* and family history, segregation data, etc. *to clinically classify* mutations *into five distinct groups as* follows: pathogenic (class5), likely pathogenic (class4), uncertain significance (class 3), likely benign (class 2), and benign (class 1). To date, only *a small percentage* of detected *mutations in the* BRCA1 *gene were clinically classified.* Therefore, there is an *urgent need in medicine to employ techniques facilitating the classification of mutations. *The use of computational approaches is a simple and cost-effective strategy for discriminating disease-associated mutations from *neutral *variants [5, 6]. 

A variety of functional assays were also developed to evaluate the consequences of mutations on protein function [-]. These methods are valuable for the classification of mutations in the *BRCA1* gene. *BRCA1* gene encodes a *1,863-aa* protein that contains two important functional domains, the highly conserved BRCT domain in the C-terminal and zinc-binding *RING finger* domain in the N-terminal [11, 12]. *Mutations in these domains were strongly associated with hereditary breast and ovarian cancers [*-*].* Currently, 108 missense mutations in the BRCT domain of *BRCA1* were reported in the BIC database (https://research.nhgri.nih.gov/bic/), but the clinical significance of only 7% of them is known. Therefore, it is *necessary to develop a method to evaluate the* pathogenicity of mutations in the BRCT domain of BRCA1. It has been shown that the BRCT domain of BRCA1 acts as a transcriptional activator when fused to the heterologous DNA binding domain. Cancer associated mutations impaired the transcription activation assay while neutral mutations displayed the activity equivalent to the wild-type BRCA1 [8, 9]. *Accordingly, a functional assay called the transcription-activation assay has been designed.* This assay is extensively validated for assessing the pathogenicity of mutations in the BRCT domain of BRCA1 [8, 9, -]*. *

In the present study, we combined transcriptional activation assay in yeast with *in silico* analysis to assess the functional impacts of two variants, As1733Gly and Val1714Gly, with unknown clinical significance in the C-terminal *of BRCA1*. These two mutations were found *in families* with hereditary breast and ovary cancers [16, 17] and reported in the BIC database. The obtained results improved the medical management of *BRCA1* *mutation* carriers.

## MATERIALS AND METHODS

**Computational analysis: **Several computational algorithms were developed for the classification of sequence variants. *These tools use different features* for prediction of disease-related *mutations such as* physicochemical properties, protein sequence, and structure. In this study, the functional effects of two variants, Asp1733Gly and Val1714Gly, were predicted using *in silico* tools Align-GVGD [18, 19], SIFT [20], Mutation Taster [21], and LRT [22]. The *details of each* method are summarized in Table 1.

**Table 1:** The details of used computational tools

**Name**	**Type**	**Deleterious**
**SIFT**	Conservation	<0.05
**Mutation Taster**	Conservation, Protein sequence annotation, Frequency	Disease causing
**LRT**	Conservation	p-value cutoff of 0.001
**Align GVGD**	Conservation, physicochemical properties	C45-C55-C65

**Yeast functional assayConstructs: **The Val1714Gly and Asp1733Gly mutations were introduced by site-directed mutagenesis with SOEing PCR [23]. Briefly, plasmid pLex9-*BRCA1* (Wild Type) (gift from Dr. *Monteiro; exons16-24*) [24] was used as a template in the first and second PCR reactions. For Val1714Gly, the first PCR was performed using the primer pairs of V1714GF/pLexR. The second PCR was performed using the primer pairs of V1714GR/pLexF. *In the case of Asp1733Gly,* two PCR fragments were amplified using primer pairs D1733GF/pLexR and D1733GR/pLexF. Finally, for each mutation, the two PCR products and primer pairs (pLexF/pLexR) were subjected to SOEing PCR, which generates a 1350 bp product. All primer sequences are *listed in* Table 2*.* For both mutations, the *PCR* fragment (1350 bp) was digested with EcoRI and BamHI enzymes, creating three fragments 928, 354 and 69 bp. The 928 bp purified fragments were subsequently cloned into the BamHI and EcoRI restriction sites of the pLex9 plasmid. Mutations were confirmed by sequencing. Plasmid constructs containing wild-type *BRCA1* (exons 16-24), as well as the neutral mutation (Ser1613Gly) and deleterious mutation (Met1775Arg) were provided by Dr. Monteiro. All plasmid constructs were confirmed by sequencing.

**Table 2:** Sequence of primers used in this study. Single nucleotide changes are underlined

**Primer**	**Sequences (** **5'→3')**
V1714GF	GAAAATGGGTAGGTAGCTATTTC
V1714GR	GAAATAGCTACCTACCCATTTT
D1733GF	GAGCATGGTTTTGAAGTCAGA
D1733GR	CTCTGACTTCAAAACCATGCTCC
pLexF	CGTCAGCAGAGCTTCACC
pLexR	TGATGTAAGCGGAGGTGTG

**Functional assay in yeast: **EGY48 strain [MATa,ura3, trp1, his3, 6 lexA operator-LEU2] [25] was transformed with the lacZ reporter plasmid pRB1840 using the lithium acetate method (Clontech). Positive colonies were selected on medium lacking uracil. The yeast cells (EGY48/pRB1840) were separately transformed with pLexA plasmid encoding wild-type *BRCA1*, Ser1613Gly, Met1775Arg, Asp1733Gly, and Val1714Gly [25, 26]. All transformations were confirmed by colony PCR and sequencing. Each variant was assayed for β-galactosidase activity *using ONPG* *[*27*]. The *experiments were carried out in triplicates. The activity was determined by comparing the results with negative (Met1775Arg) and positive (wild-type *BRCA1* and Ser1613Gly) controls.

## RESULTS

In present study, different computational algorithms, including Align-GVGD [18, 19], SIFT [20], Mutation Taster [21], and LRT [22] were utilized for classifying SNPs. SIFT and LRT used sequence homology, Align GVGD combines evolutionary conservation and biophysical characteristics of amino acids, and Mutation Taster used from a subset of evolutionary conservation, mutation frequency and protein sequence annotations for prediction of *the impact of mutations* on protein function. Computational tools SIFT, Align-GVGD, Mutation Taster, and LRT predict the Val1714Gly as deleterious, while Asp1733Gly, was predicted to be neutral by all computational tools except SIFT (Table 3).

**Table 3:** Predicted effects of variants by computational tools

**Mutations**	**SIFT** Score (interpretation)	**Mutation Taster** Interpretation	**LRT** Score (interpretation)	**LRT** Score (interpretation)
**Asp1733Gly**	0.01 (Neutral)	Disease causing	0.037 (Neutral)	C0 (Neutral)
**Val1714Gly**	0 (Damaging)	Disease causing	0 (Deleterious)	C65 (Deleterious)

**Figure 1:**
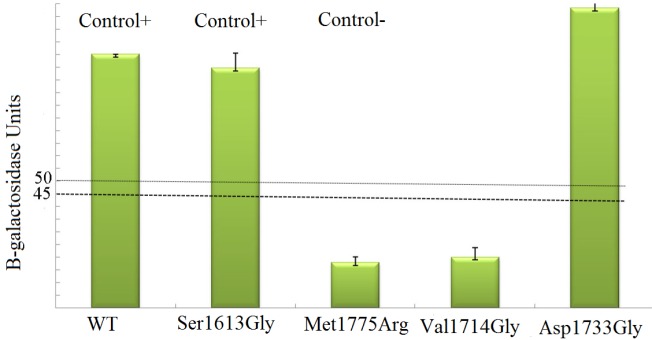
Transcriptional assay of BRCA1.

To determine transcriptional activation of the BRCT domain, wild-type or neutral variant Ser1613Gly were used as positive controls and *cancer-derived mutation *Met1775Arg *was considered as the negative control [*8*, *9*, *28*]. Our findings showed that the wild-type BRCA1 significantly activated transcription in the yeast system. *The positive control Ser1613Gly showed activity similar *to the wild-type* protein, whereas the Met1775Arg loses *its ability to* activate *transcription (*Fig. 1*).* To examine the functional consequences of Val1714Gly and Asp1733Gy mutations, we compared their ability to activate the lacZ reporter with positive and negative controls. It has been shown that variants with more than 50% wild- type activity were considered as neutral and those with less than 45% wild-type activity as deleterious [14]. The Asp1733Gly displayed activity slightly higher than wild-type, whereas Val1714Gly showed <20% of the wild type activity in yeast. Using the threshold defined in the present study, Asp 1733Gly and Val1714Gly can be classified as benign and pathogenic, respectively.

Wild-type BRCA1 and Ser1613Gly neutral variant were used as positive controls, and deleterious variant Met1775Arg was used as the negative control. The positive control Ser163Gly revealed activity similar *to the wild-type,* whereas the Met1775Arg mutation *lost its ability to* activate *transcription.* Variants with more than 50% wild- type activity were considered as neutral and those with less than 45% wild-type activity as deleterious Val1714Gly considerably *reduced* the *activity in yeast* (~20% of wt), whereas the Asp1733Gly variant displayed *activity* slightly higher than the wild-type.

## DISCUSSION

In the present study, a combination of *in silico* tools and *transcriptional activation assay in yeast* were used to assess the functional consequences of the two variants, Asp1733Gly and Val1714Gly, in the C-terminal *of BRCA1*. These two mutations were found *in families* with hereditary breast and ovary cancers [16, 17] and have not been classified by the IARC *BRCA *expert panel. 

*Nowadays,* a variety of functional assays including small colony phenotype assay, rescue of radiation resistance, ubiquitin ligase activity, and transcriptional activation assay were developed to evaluate the impacts of mutations on BRCA1 function [-]. Transcription assay is perhaps the *most widely used assay for BRCT domain integrity of BRCA1. *This assay was performed in both yeast and mammalian cells. In most cases, a significant correlation was *observed between* functional assay in yeast and mammalian cells. *However, Vallon-Christersson et al. reported a* discrepancy between results from the *transcriptional activity of* R1699W variant in yeast and mammalian cells [13]. On the other hand, it is important to carry out the parallel *yeast-based transcription assays* because multiple mutant transcripts showed reduced expression of BRCA1 protein, suggesting instability of the protein product in mammalian cells [10, 14]. These observations raise the question of whether the variant is truly pathogenic if the results were normalized to *expression levels of BRCA1 protein* in mammalian cells.

In the present study, the functional assay results showed that the Asp1733Gly has comparable activity to the wild-type, while Val1714Gly mutation exhibit significantly decreased activity in yeast. This result is consistent with the previous study that investigated the effects of mutations by transcriptional assays in mammalian cells [15] . Also, our computational analysis showed that the Val1714Gly mutation was deleterious, while the other one, Asp1733Gly, predicted as neutral. These results are consistent with the obtained data from the functional assay in yeast. *The combinations of obtained results from all functional assays and computational tools provide* strong evidence for or *against the pathogenicity of variants of uncertain significance.* So, our findings *enhance the possibility* that Asp1733Gly and Val1714Gly are benign and pathogenic, respectively.
